# Reactive oxygen species scavenging mechanisms associated with polyethylene glycol mediated osmotic stress tolerance in Chinese potato

**DOI:** 10.1038/s41598-020-62317-z

**Published:** 2020-03-25

**Authors:** Manas Ranjan Sahoo, Tongbram Roshni Devi, Madhumita Dasgupta, Potshangbam Nongdam, Narendra Prakash

**Affiliations:** 1 ICAR Research Complex for North Eastern Hill Region, Imphal, 795004 Manipur India; 2 Manipur University, Canchipur, Imphal, 795003 Manipur India

**Keywords:** Environmental biotechnology, Abiotic

## Abstract

Influence of polyethylene glycol (PEG) mediated osmotic stress on reactive oxygen species (ROS) scavenging machinery of Chinese potato (*Solenostemon rotundifolius* (Poir.) J. K. Morton) was investigated. Five genotypes of Chinese potato were raised in Murashige and Skoog (MS) basal medium containing 6–benzylaminopurine (BAP, 1 mg L^–1^) along with various concentrations of PEG–6000 mediated stress conditions (0, –0.2 and –0.5 MPa) and evaluated for osmotic stress tolerance *in vitro*. The medium containing PEG–6000 had a detrimental effect on plantlet growth and development while compared with the control. Accumulation of H_2_O_2_ was lower in Sreedhara and Subala and higher in Nidhi under PEG stress, which was evident by *in situ* detection in leaves. Lipid peroxidation product such as malondialdehyde (MDA) content was increased due to PEG stress which was more in susceptible genotype than that in tolerant ones. An enhanced ROS–scavenging antioxidant enzyme was observed under stress with respect to the control. The enzymes of ascorbate–glutathione cycle showed an important role in scavenging ROS. The imposition of PEG stress also increased the non–enzymatic antioxidants *viz*., the ascorbate and reduced glutathione content which was prominent in tolerant genotypes in comparison to susceptible. The present study indicated that, Sreedhara and Subala showed more tolerance to osmotic stress with better ROS scavenging machineries which would be the lines of interest for augmenting future breeding strategies in this climate resilient minor tuber crop.

## Introduction

Chinese potato (*Solenostemon rotundifolius* (Poir.) J. K. Morton), belongs to the family Lamiaceae, is one of the important minor tuber crops grown in the tropics of the world. The tubers of this crop are rich in carbohydrates and minerals^1^ which provide essential dietary and energy supplements during the lean periods. It could be the better alternative to potato (*Solanum tuberosum* L.) for the tropics of the world under changing climatic conditions; where potato fails to grow due to global warming. Chinese potato can grow in a wide range of environmental conditions. Despite its wide adaptability, the productivity (18–20 t/ha) was severely affected due to osmotic stress.

Under natural conditions, plant growth and development often challenged by various stresses^2^, of which, osmotic stress is one of the most limiting factors which can cause 20–98% of yield reductions^3^. To elucidate the plant response to osmotic stress, *in vitro* cultures are preferred as it minimizes nutrient and environmental variations under control conditions^4^. In this investigation, we have used polyethylene glycol (PEG–6000) to induce osmotic stress conditions *in vitro*. In plant osmotic stress studies, PEG is widely used as a potential osmoticum in the nutrient medium to induce water deficit^5^.

Induced osmotic stress resulted in overproduction of reactive oxygen species (ROS) which was considered as a hallmark of plant stress response. To scavenge the toxic consequences of ROS, plant deploys antioxidative mechanisms^6^. Oxidative burst led superoxide radicals are dismutated into H_2_O_2_ by superoxide dismutase (SOD). The liberated H_2_O_2_ is further scavenged by catalase (CAT) and guaiacol peroxidases (GPX). A major H_2_O_2_ detoxifying system operating in plants is also associated with ‘ascorbate–glutathione cycle’ (ASA–GSH) consisting of four enzymatic antioxidative machineries. In this cycle, ascorbate peroxidase (APX) efficiently manages H_2_O_2_ during stress than CAT. It uses ascorbic acid and oxidizes it to monodehydro ascorbate (MDAR); which gets reduced to regenerate the ascorbate pool^7^. Similarly, glutathione reductase (GR) converts oxidised glutathione (GSSG) to reduced glutathione (GSH)^8^.

Synthesis of non–enzymatic antioxidants *viz*., phenolic compounds, ascorbic acid (ASA) and reduced glutathione (GSH) also act as a protective mechanism against oxidative burst. Malondialdehyde (MDA) is one of the lipid peroxidation products indicates ROS induced oxidative damage in plant tissues^9^. The peroxidation of membrane lipids and its role in osmotic stress tolerance in Chinese potato is still unexplored.

Understanding physiological and biochemical mechanisms and ROS scavenging machinery would shed light on inherent stress tolerance potential of plant against adverse conditions^10^. The present study was carried out to investigate ROS scavenging mechanisms in five genotypes of Chinese potato (*Solenostemon rotundifolious* (Poir.) J. K. Morton) under *in vitro* PEG mediated oxidative stress conditions.

## Materials and methods

### Experimental site and plant materials

This study was carried out at Indian Council of Agriculture Research (ICAR) Research Complex for North Eastern Hill Region, Manipur Centre, Imphal, India during 2015–16. Five genotypes of Chinese potato *viz*., Sreedhara, Subala, Nidhi, TVM and CO–1 were used as the source materials for ROS scavenging mechanisms studies under PEG mediated osmotic stress conditions.

### Explants, growth conditions, treatments and growth parameters

Nodal explants (5–6 mm) of the five genotypes of Chinese potato were collected from poly house and pre–treated with fungicide (Carbendazim 0.15%) for 10 min. The explants were cleaned thoroughly in running tap water, surface sterilized in 2% sodium hypochlorite solution for 3 min. and rinsed thrice in sterile water. Culture medium included Murashige and Skoog^11^ basal medium with 6–benzylaminopurine (BAP, 1 mg L^–1^) and sucrose (30 g L^–1^) solidified with phytagel (3 g L^–1^). PEG–6000 at a concentration of 118.0 and 197.0 g L^–1^ was incorporated in MS medium to induce osmotic stress of –0.2 and –0.5 MPa, respectively and pH was adjusted to 5.8 ± 1. Equal amount of culture media (15 ml) was poured into the test tubes (25 × 100 mm, Borosil, India) and sterilized in a steam autoclave (Remi, India) at 105 kPa for 15 min. Nodal explants of all the five genotypes were inoculated in the MS medium with different levels of PEG. The explants inoculated in MS + BAP alone were maintained as the control. All cultures were incubated at 25 ± 2 °C with 16 h photoperiod and 40 µmol m^–2^ s^–1^ fluorescence light (Phillips, India) for 6 weeks. The experiment was set up in a 5×3 factorial completely randomized design (fCRD) replicated thrice with triplicate determinations. Shoot and root proliferation of *in vitro* plantlets were observed at 6 weeks of inoculation.

### Estimation of H_2_O_2_, *in situ* detection of H_2_O_2_ and lipid peroxidation

H_2_O_2_ content in leaf tissues of Chinese potato under different treatments was estimated following the method of Velikova *et al.*^12^ by measuring the oxidation product at 390 nm. H_2_O_2_ was estimated using standard concentrations of 0–100 µM and was expressed as µM g^–1^ FW.

H_2_O_2_ produces a brown stain in plant tissues which is localized by 3,3–diaminobenzidine (DAB)^13^. Vacuum infiltration of whole leaves was done with DAB solution (1 mg mL^–1^) followed by incubation at dark for 12–16 hrs with mild agitation (80–100 rpm) in a rotating shaker (Tarson, India). The chlorophyll was completely removed from the leaves by treating with ethanol:acetic acid:glycerol (3:1:1) and incubated in the circulating water bath (Thermo Fisher Scientific, USA) at 75 °C. The DAB polymerized reddish–brown spots produced on site of the leaf tissues was photographed by a 10 mega prixel digital camera (Sony, India).

The cellular damage caused by ROS can be estimated by lipid peroxidation of the cell membrane; which is measured in terms of malondialdehyde (MDA) content^14^. MDA content (nM g^–1^ FW) in the leaves was determined using the extinction coefficient^15^ of 155 mM^–1^ cm^–1^.

### Determination of antioxidant enzymatic activity

Enzymatic antioxidants (SOD, CAT, GPX, APX, MDAR, DHAR and GR) were estimated from six weeks old *in vitro* leaf tissues (0.25 g). The leaf samples were ground into powder using liquid nitrogen and homogenised with 50 mM of extraction buffer (NaPO_4_, pH 7.8) containing ethylenediamine tetra acetic acid (EDTA, 1 mM), Triton X–100 (0.1%), ascorbate (1 mM) and sorbitol (10%) and then centrifuged (15,000 rpm for 20 min) at 4 °C. The supernatants were collected for estimation of antioxidative enzymes.

SOD activities (EC 1.15.1.1) was estimated by measuring the inhibition ability of the nitroblue tetrazolium chloride (NBT) reactions^16^. The unit of SOD enzyme inhibiting 50% NBT was expressed as U g^–1^ FW of leaf sample. Similarly, CAT activity (EC 1.11.1.6) was determined as the rate of H_2_O_2_ scavenged which was measured by decrease in absorbance at 240 nm. Quantification of CAT activity was performed by its molar extinction coefficient (40 mM^–1^ cm^–1^) following the method of Aebi^17^.

GPX activity (EC 1.11.1.7) was estimated observing the production of tetraguaiacol using extinction coefficient (26.6 mM^–1^ cm^–1^)^18^. APX activity (EC 1.11.1.1) was assayed by observing the decline in absorbance at 290 nm due to oxidation of ascorbate and quantified using molar extinction coefficient (2.8 mM^–1^ cm^–1^)^19^. MDAR (EC 1.6.5.4) was determined according to the method derived by Hossain and Asada^20^ and the activity was observed by reduction in absorbance at 340 nm for 1 min using extinction coefficient^21^ of 6.22 mM^–1^ cm^–1^. DHAR (EC 1.8.5.1) catalyses oxidised ascorbate to ascorbate. Activity of DHAR was measured using extinction coefficient (2.8 mM^–1^ cm^–1^)^19^. GR (EC 1.6.4.2) activity was measured following the rate of oxidation of NADPH and quantified using extinction coefficient (6.22 mM^–1^ cm^–1^)^22^. All the enzymatic assays were carried out by reading the absorbance using a UV–visible spectrophotometer (Thermo Fisher Scientific, USA).

### Determination of non–enzymatic antioxidants: ascorbate (ASA) and reduced glutathione (GSH) content

Ascorbate content (ASA) was estimated following a standard curve plotted with known concentrations of ascorbate in leaf tissue and expressed as mg g^–1^ FW^23^. GSH was estimated following standard method^24^ and quantified using standard concentrations of reduced glutathione (µmol g^–1^ FW).

### Statistical analysis

Data on *in vitro* growth parameters, biochemical estimations and ROS scavenging antioxidants were recorded at six weeks of inoculation under control and PEG treatments (–0.2 and –0.5 MPa). The experiments were conducted in 5 × 3 factorial completely randomized design (fCRD). Analysis of variance (ANOVA)^25^ was performed to test the significance at probability level *P* ≤ 0.05 and *P* ≤ 0.01. Tukey’s test was performed to compare significant differences among the mean values^26^. Graphical representations are mean of three replications with triplicate determinations.

## Results

### Influence of PEG mediated osmotic stress on *in vitro* growth responses of Chinese potato

**Figure 1:**
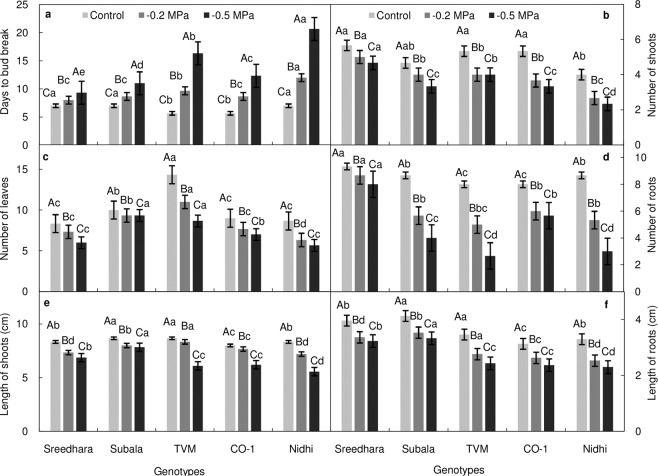
(**a–f**) Effect of *in vitro* PEG mediated osmotic stress on growth response of Chinese potato genotypes. (**a**) days to bud break, (**b**) number of shoots, (**c**) number of leaves, (**d**) number of roots, (**e**) length of shoots (cm) and (**f**) length of roots (cm). Values are the mean of three replicates and bars represent standard error of means. Different letters in upper case represent significant differences between the treatments (control, –0.2 MPa and –0.5 MPa) in the genotypes and lower case represents significant difference among the genotypes under each treatment according to *Tukey’s* test.

Analysis of variance for *in vitro* growth parameters (Table 1) showed significant effects across the genotypes and PEG treatments. Growth of *in vitro* plantlets decreased significantly under PEG treatments (–0.2 and –0.5 MPa) while compared with the stress–free control. Days to bud break was severely delayed in response to PEG stress (Fig. 1a). After 6 weeks of culture, decrease in number of shoots, leaves, and roots, length of shoots and roots were more pronounced in the higher osmotic treatment (–0.5 MPa) (Fig. 1b–f, *P* ≤ 0.01). Sreedhara and Subala demonstrated less variation in growth parameters than other genotypes under moisture stress as compared to control.

**Table 1:** Two way analysis of variance for growth parameters of Chinese potato genotypes under *in vitro* PEG mediated osmotic stress conditions in a 5 × 3 factorial experiment in complete randomized design (fCRD).

Source	*df*	Days to sprout	No. of shoots	No. of leaves	No. of roots	Length of shoots	Length of roots
Genotypes	4	37.6**	5.3**	30.9**	16.1**	1.7*	1.90**
PEG	2	212.3**	8.9**	28.7**	57.2**	13.7**	3.02**
Genotypes × PEG	8	17.0**	0.2^NS^	2.7^NS^	2.9*	0.8^NS^	0.02^NS^
Error	30	5.7	1.3	4.7	1.2	0.4	0.2

*^,^ ** and ^NS^ Indicates *p *≤ *0.05*, *p *≤ *0.01* and *p *>* 0.05*, respectively.

### Influence of PEG stress on H_2_O_2_ production, *in situ* detection and lipid peroxidation of Chinese potato

**Figure 2:**
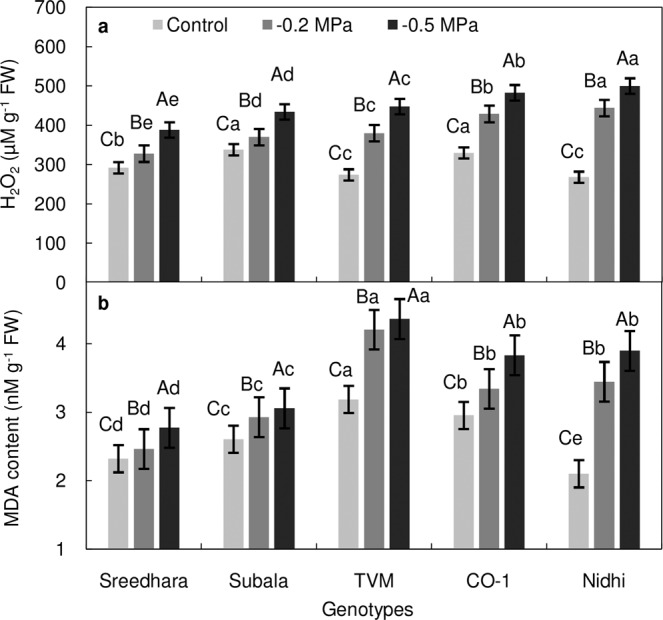
(**a–b**) Effect of *in vitro* PEG mediated osmotic stress on H_2_O_2_ production (**a**) and lipid peroxidation (**b**) in leaf tissues of Chinese potato genotypes. Values are the mean of three replicates and bars represent standard error of means. Different letters in upper case represent significant differences between the treatments (control, –0.2 MPa and –0.5 MPa) in the genotypes and lower case represents significant difference among the genotypes under each treatment according to *Tukey’s* test.

**Figure 3:**
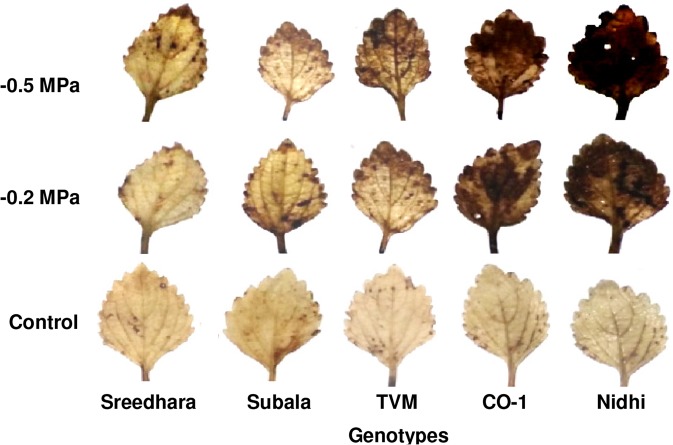
Effect of *in vitro* PEG mediated osmotic stress on *in situ* detection of ROS in leaf tissues of Chinese potato genotypes.

ANOVA for H_2_O_2_ accumulation and lipid peroxidation (MDA content) revealed significant variation under PEG induced osmotic stress, genotypes and genotype x PEG interactions at *P* ≤ 0.01 (Table 2). H_2_O_2_ activity in leaf tissues of Chinese potato was increased with increase in PEG stress (Fig. 2a). Figure 2b represents lipid peroxidation (MDA content) in leaf tissues of control and PEG treated Chinese potato. A significant increment in MDA content was observed among the studied genotypes; which was more in Nidhi than Sreedhara and Subala (Fig. 2b). Figure 3 depicts the *in situ* detection of ROS through DAB staining in Chinese potato leaves. The PEG treated leaves turned to dark brown which was more pronounced in Nidhi than in Sreedhara and Subala. As expected, the control leaves showed no brown precipitation (Fig. 3).

**Table 2:** Two way analysis of variance for biochemical estimations and ROS scavenging antioxidants of Chinese potato genotypes under *in vitro* PEG mediated osmotic stress conditions in a 5 × 3 factorial experiment in complete randomised design (fCRD).

Source	Genotypes (G)	PEG (P)	GxP	Error
*df*	4	2	8	30
H_2_O_2_	5742.1**	57120.4**	2273.7*	582.5
MDA	2.621**	3.670**	0.315^NS^	0.222
SOD	495.6**	4103.1**	188.9**	34.2
CAT	830.8**	1899.9**	98.5^NS^	126.2
GPX	15966.7**	60402.5**	4789.3**	17.4
APX	48852.0**	46216.0**	4336.7^NS^	1743.2
MDAR	1920.7**	3494.6**	307.8^NS^	312.2
DHAR	283949.8**	80782.3**	13127.1**	3231.3
GR	17702.1**	12478.9**	2469.4**	609.7
Ascorbate	1.176**	1.864**	0.056^NS^	0.029
Reduced glutathione	1.236**	36.178**	3.681**	0.008

*^,^ ** and ^NS^ Indicates *p *≤ *0.05*, *p* ≤ *0.01* and *p *>* 0.05*, respectively.

### Influence of PEG stress on antioxidative enzymes activities of Chinese potato

**Figure 4:**
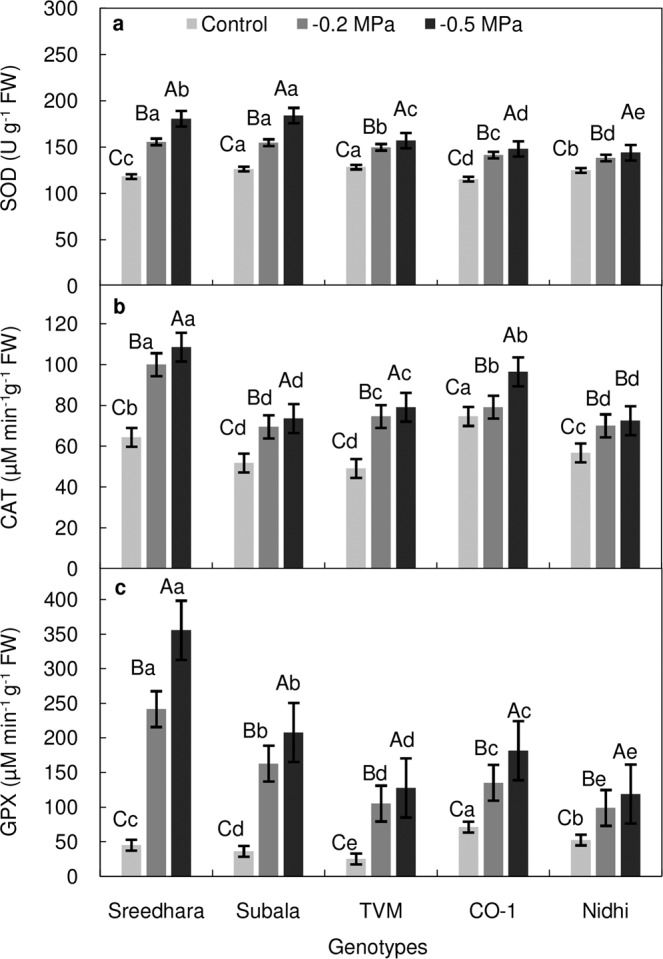
(**a–c)** Effect of *in vitro* PEG mediated osmotic stress on antioxidative enzyme activities of leaf tissues of Chinese potato genotypes. (**a**) superoxide dismutase (SOD, U g^–1^ FW), (**b**) catalase (CAT, µM min^–1^g^–1^ FW) and (**c**) guaiacol peroxidase (GPX, µM min^–1^g^–1^ FW), Values are the mean of three replicates and bars represent standard error of means. Different letters in upper case represent significant differences between the treatments (control, –0.2 MPa and –0.5 MPa) in the genotypes and lower case represents significant difference among the genotypes under each treatment according to *Tukey’s* test.

ANOVA showed significant differences (*P* ≤ 0.01) between the activity levels of ROS scavenging antioxidative enzymes under PEG mediated osmotic stress (Table 2). The activity of all seven antioxidative enzymes increased in Chinese potato leaves when exposed to varying concentrations of PEG stress (Fig. 4a–c). The SOD activity was increased steeply in genotypes Sreedhara and Subala under stress conditions as compared to control (Fig. 4a). Followed by this, the CAT activity showed the similar increasing trend with the increased osmotic stress level (Fig. 4b). The magnitude of increase in CAT activity was highest in Sreedhara (68.50%), as compared to Nidhi (29.25%) under higher stress conditions over control. The activity of GPX also significantly increased upon PEG stress (Fig. 4c), being more pronounced in Sreedhara and Subala compared to Nidhi and CO–1.

### Influence of PEG stress on ASA–GSH cycle of Chinese potato

**Figure 5:**
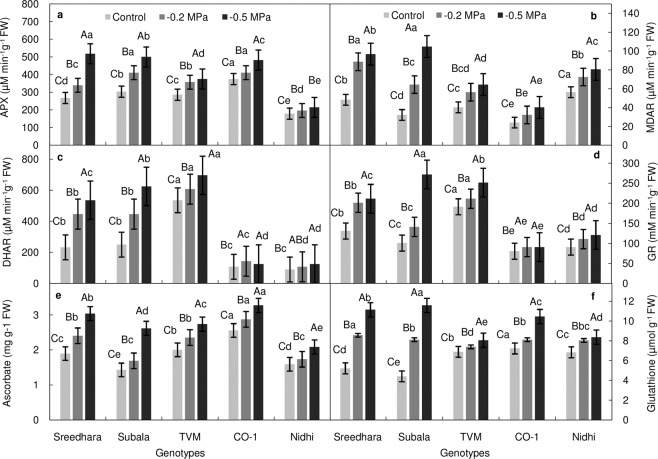
(**a–f)** Effect of *in vitro* PEG mediated osmotic stress on ASA–GSH cycle of Chinese potato genotypes. (**a**) ascorbate peroxidase (APX, µM min^–1^g^–1^ FW), (**b**) monodehydro ascorbate reductase (MDAR, µM min^–1^g^–1^ FW), (**c**) dehydro ascorbate reductase (DHAR, µM min^–1^g^–1^ FW), (**d**) glutathione reductase (GR, mM min^–1^g^–1^ FW), (**e**) ascorbate (ASA, mg g^–1^ FW), and (**f**) reduced glutathione (GSH, µmol g^–1^ FW). Values are the mean of three replicates and bars represent standard error of means. Different letters in upper case represent significant differences between the treatments (control, –0.2 MPa and –0.5 MPa) in the genotypes and lower case represents significant difference among the genotypes under each treatment according to *Tukey’s* test.

The enzymes of ASA–GSH cycle also showed an increment in their activities when imposed to PEG mediated osmotic stress (Fig. 5a–d). Subala registered significantly higher induction of all four enzymes of this cycle *viz*., APX, MDAR, DHAR and GR; whereas, Nidhi showed the least induction in the similar conditions.

Following PEG imposed stress in leaves, ascorbate (Fig. 5e) and reduced glutathione (Fig. 5f) was increased significantly in all the tested genotypes with respect to their control. Ascorbate content under osmotic stress (–0.5 MPa) was increased to the tune of 83.04% in Subala followed by 60.08% in Sreedhara; the same was lower in Nidhi in all the treatments while compared with other genotypes (Fig. 5e). In a similar way, reduced glutathione was increased by 1.63 and 1.14–fold in Subala and Sreedhara, respectively at higher PEG stress as compared to their controls (Fig. 5f).

Overall result implied that the genotypes Sreedhara and Subala performed better under induced PEG stress with the lower accumulation of H_2_O_2_ and MDA which further impaired by induction of various antioxidants (enzymatic and non–enzymatic) as the powerful scavengers of ROS. TVM and CO–1 showed moderately susceptible and Nidhi was characterized as susceptible to moisture stress.

## Discussion

We have taken an *in vitro* approach to study the effect of PEG mediated osmotic stress on ROS–scavenging mechanisms among five genotypes of Chinese potato. *In vitro* evaluation is an efficient and quick approach for understanding stress tolerance which showed the similar effect as compared to the labour–intensive field–based screening^27^. So far, there is no report available on effects of PEG mediated osmotic stress on growth responses of Chinese potato *in vitro*. PEG at high molecular weight acts as a non–intrusive osmotic agent, that overcast the water potential in the culture media and thereby, widely used in osmotic stress tolerance studies in plants^28^. Growth responses of nodal explants of Chinese potato significantly decreased when PEG was incorporated in MS medium. The first detrimental effect of PEG in the shoot multiplication media was evident by a gradual delay in bud break from lower to higher stress as compared to the control. The bud break was earlier by 10 days of inoculation in Sreedhara and Subala, whereas, it was delayed by 3 weeks in Nidhi at –0.5 MPa stress. The overall growth in terms of number of leaves, number of shoots, length of shoots and rooting was highly affected due to PEG imposed media as compared to the control. Under PEG stress condition, Subala and Sreedhara maintained the growth by registering up to 20% decrease in shoot proliferation and rooting, which reflects its inherent tolerance towards osmotic stress. On the contrary, Nidhi showed an indication of susceptibility towards PEG stress by exhibiting more growth retardation *in vitro*. Our previous studies in taro, a carbohydrate–rich tuber crop, demonstrated the detrimental effect of PEG on shoot proliferation *in vitro*^29^. Reports have also shown that limited water availability restricted cell division and elongation and thereby hinders plant growth and development^30,31^. The extent of plant growth inhibition depends on the genotype and the severity of stress; which was prominent in our study across the studied genotypes.

ROS generated inside the cell as a result of oxidative stress can target the lipid membranes resulting in elicitation of the lipid peroxidation^31,32^. Increased MDA content is considered as a hallmark of membrane lipid peroxidation and also an indicator of free radical prevalence in the tissues. To understand whether PEG mediated osmotic stress results in the induction of ROS in Chinese potato, we have measured the level of H_2_O_2_ and MDA content in control and stressed leaves. Our results have shown a significant increment in both H_2_O_2_ and MDA content; which shows the involvement of oxidative stress incurred by imposing to PEG stress among the studied genotypes. Sreedhara and Subala registered lower H_2_O_2_ and MDA accumulation (up to 20%) at higher PEG (–0.5 MPa) as a sign of lower osmotic imbalance. However, Nidhi possessed higher osmotic imbalance with higher accumulation of H_2_O_2_ and MDA (up to 85%) than its control. In support of our results, Fu *et al*.^33^ have shown that MDA content of cassava exhibited a linear change under higher PEG treatments. Studies have shown that MDA content and H_2_O_2_ accumulation was about two to four–fold in susceptible genotypes than the tolerant ones^34–36^. From our studies, it was evident that Sreedhara and Subala maintained lower H_2_O_2_ and MDA content irrespective of PEG doses which reflect more membrane stability and also an indication of early defense to combat osmotic stress imposed by PEG.

To further confirm, we have also carried out *in situ* detection of H_2_O_2_ in leaves by DAB staining. H_2_O_2_ along with peroxidases (a haem–containing protein) oxidizes DAB, which is visualized by a dark brown precipitation in the leaves. Among the genotypes, Nidhi followed by CO–1 showed extensive dark stained DAB sites in the leaves making the leaves turning completely black which is correlated with higher accumulation of H_2_O_2_ compared to Subala and Sreedhara (where only leaf edges were stained). Chakraborty and Pradhan^35^ documented higher accumulation of H_2_O_2_ was associated with the appearance of darkly stained DAB spots in leaves of susceptible varieties of wheat than the tolerant ones; which is in support of our results. Oxidative burst in leaf tissues through formations of free radicals due to PEG stress was characterized by increased ROS production as reflected by a sharp increase in H_2_O_2_ production and also, increased level of MDA. This holds true in the *in situ* DAB test for ROS generation; where the more extensive brown stain was detected in Nidhi leaves than that of Subala and Sreedhara.

The ability of the fast and effective antioxidant response in stress situations can reflect the stress tolerance ability of the plant^37^. In our study, activities of all antioxidative enzymes were significantly increased under osmotic stress across the Chinese potato genotypes which were higher in Subala and Sreedhara as compared to other genotypes. Khanna–Chopra and Selote^38^ reported a higher level of antioxidative machineries in stressed plants over non–stressed plants. Similarly, Wang *et al*.^39^ reported that the osmotic adjustment ability in tolerant cultivar is more efficient over susceptible ones. SOD is the first line of antioxidant which rapidly scavenges O_2_^–^ and restricts the production of OH^40^. We have observed upto 52.6% increase in the activity of SOD in Sreedhara followed by Subala (45.6%) over control, suggesting that these genotypes have inherent O_2_^.–^ radical scavenging ability than other studied genotypes (Nidhi, 15.24%).

H_2_O_2_ produced is further reduced to H_2_O by two major antioxidative enzymes –CAT and GPX. CAT activity increased with increase in PEG stress and was found to be 1.7–fold induced in Sreedhara at higher stress conditions; showing its efficiency in reducing the toxic effects of generated H_2_O_2_. Our previous study also showed an increase in CAT activity under PEG induced osmotic stress in taro^29^. We have also observed a significant increment in the activity of H_2_O_2_ scavenging enzyme GPX in Sreedhara (upto 7.8–fold change) at higher stress. This can be correlated with *in situ* DAB staining data; where very less brown stain was observed in Sreedhara leaves restricted at the edges only. This enhanced activity could explain the detoxification of H_2_O_2_ mainly occurred through GPX than CAT. Therefore, from our results it is apparent that GPX plays a stringent role in H_2_O_2_ elimination than CAT in Chinese potato. The genotype Sreedhara performed better by registering maximum fold change in these enzyme activities. Previous report showed enhanced activity of GPX was greatly associated with prolonged drought spell in wheat varieties^35^.

Our results also showed that ascorbate–glutathione cycle (ASA–GSH) played an important role on scavenging the over production of H_2_O_2_ due to osmotic stress in Chinese potato genotypes. APX, GR, MDAR and DHAR involved in ASA–GSH cycle were also higher in stressed plants than that in control^39,41^. This series of enzymatic and non–enzymatic antioxidants such as SOD, CAT, GPX and DHAR delays leaf senescence induced by PEG stress^42^.

ASA acts as an important defensive antioxidant against overproduction of ROS induced by oxidative stress^43,44^. Resistant varieties maintained higher ascorbate content over susceptible ones under different levels of drought stress^39^. This antioxidant plays a major role either in preventing or in lowering the risk caused by ROS in higher plants; by removal of H_2_O_2_ *via* ASA–GSH cycle. Reduced glutathione also participated in ROS inhibition and induced drought stress in *Arabidopsis* plants^45^. In our study, the magnitude of increase in these two antioxidants was lower in Nidhi than Subala and Sreedhara; showing the inability of Nidhi to cope up with induced oxidative stress and making it as susceptible.

## Conclusions

*In vitro* PEG mediated osmotic stress resulted in oxidative burst as evident by accumulation of H_2_O_2_ and lipid peroxidation. Increment in enzymatic and non–enzymatic antioxidants under stress implied a positive hallmark of stress tolerance in the present study. Enhancement in the accumulation of ascorbic acid and reduced glutathione accompanied by increased GR activity depicts the involvement of ASA–GSH cycle in osmotic stress tolerance in Chinese potato. Of the five genotypes tested, Sreedhara and Subala exhibited distinct antioxidative mechanisms for scavenging of ROS and protecting the plants from deleterious effects of PEG mediated osmotic stress. Considering all the above data, our study revealed that Sreedhara and Subala were tolerant, TVM and CO–1 were moderately tolerant and Nidhi was susceptible to PEG stress. The detailed information on ROS scavenging machineries would be useful for intensification of breeding strategies in Chinese potato for development of tolerant lines to harsh environments.
